# Ultradeep 16S rRNA Sequencing Analysis of Geographically Similar but Diverse Unexplored Marine Samples Reveal Varied Bacterial Community Composition

**DOI:** 10.1371/journal.pone.0076724

**Published:** 2013-10-22

**Authors:** Chairmandurai Aravindraja, Dharmaprakash Viszwapriya, Shunmugiah Karutha Pandian

**Affiliations:** Department of Biotechnology, Alagappa University, Karaikudi, Tamil Nadu, India; J. Craig Venter Institute, United States of America

## Abstract

**Background:**

Bacterial community composition in the marine environment differs from one geographical location to another. Reports that delineate the bacterial diversity of different marine samples from geographically similar location are limited. The present study aims to understand whether the bacterial community compositions from different marine samples harbour similar bacterial diversity since these are geographically related to each other.

**Methods and Principal Findings:**

In the present study, 16S rRNA deep sequencing analysis targeting V3 region was performed using Illumina bar coded sequencing. A total of 22.44 million paired end reads were obtained from the metagenomic DNA of Marine sediment, Rhizosphere sediment, Seawater and the epibacterial DNA of Seaweed and Seagrass. Diversity index analysis revealed that Marine sediment has the highest bacterial diversity and the least bacterial diversity was observed in Rhizosphere sediment. Proteobacteria, Actinobacteria and Bacteroidetes were the dominant taxa present in all the marine samples. Nearly 62–71% of rare species were identified in all the samples and most of these rare species were unique to a particular sample. Further taxonomic assignment at the phylum and genus level revealed that the bacterial community compositions differ among the samples.

**Conclusion:**

This is the first report that supports the fact that, bacterial community composition is specific for specific samples irrespective of its similar geographical location. Existence of specific bacterial community for each sample may drive overall difference in bacterial structural composition of each sample. Further studies like whole metagenomic sequencing will throw more insights to the key stone players and its interconnecting metabolic pathways. In addition, this is one of the very few reports that depicts the unexplored bacterial diversity of marine samples (Marine sediment, Rhizosphere sediment, Seawater) and the host associated marine samples (Seaweed and Seagrass) at higher depths from uncharacterised coastal region of Palk Bay, India using next generation sequencing technology.

## Introduction

Marine environment, which constitutes the largest portion of earth, comprises highly diverse and complex microbial communities. Marine bacteria are important for maintenance of carbon dynamics in marine ecosystem. The presence of variety of heterotrophic bacteria and their importance is very well recognized for sustained ecological and biogeochemical cycle in marine environment [1]–[3].

Both culture dependent and culture independent methods have been widely deployed to characterize heterotrophic bacterial composition and its distribution in marine ecosystem. Since culture dependent methods fail to delineate the true bacterial diversity in the given environment, culture independent methods including T-RFLP, SSCP and DGGE have been widely used to study the bacterial diversity [4]–[6]. Though these techniques provide a bird's eye view of bacterial community composition, ultra deep sequencing using next generation technology revolutionized our understanding of complex microbial community in a broader way.

Availability of unprecedented 16S rRNA gene sequences in public databases provides a testament to the deployment of Next generation sequencing technology in studying bacterial diversity. Though 454 (Roche) had been widely utilized to study the bacterial diversity, low sequencing cost, high depth coverage of Illumina (Solexa) sequencing technology has surpassed the utility of 454 in microbial diversity analysis [7]–[9]. In addition, enhanced sample throughput using bar coding, advancement in Illumina sequencing platform (MiSeq, HiSeq) provide a pre-eminent view of microbial composition than analyzed with other sequencing technologies [10]. Unprecedented data using Illumina with high taxonomic resolution gain insights to understand even the rare microbes in different environmental habitats [11], [12].

Plethora of information is available about the bacterial community composition of different marine habitats along the coastal regions of western countries [13]–[16]. Few reports are available about the bacterial diversity along the coastal region in India. Lack of in-depth analysis of bacterial diversity were observed in these studies as most of the works were restricted based on culture dependent and other low throughput techniques [6], [17], [18]. Hence a considerable effort has been taken in the present study to explore variability in bacterial community composition in hitherto uncharacterized geographically related marine environmental samples through high throughput sequencing. Comparative assessment of heterotrophic bacterial community of these marine samples were carried out using bar-coded Illumina sequencing of hypervariable V3 region of 16S rRNA gene. This study will ameliorate our understanding of the variation in distribution of microbial assemblages in these geographically related marine samples in a better way and also throw more insight to the biotechnological potential of previously unexplored marine habitats.

## Results and Discussion

### Generation of multimillion Illumina reads and production of high quality reads

To delineate the bacterial community composition among the geographically linked marine samples, we carried out high throughput Illumina paired end sequencing. Rhizosphere sediment (*Rhizophora apiculata*), marine sediment, seawater, seaweed (*Gracilaria* sp.) and seagrass (*Cymodacea* sp.) of Karankadu coastal region, Palk Bay, India are the five different geographically linked marine samples chosen for the present study. The bacterial diversity in Palk Bay is hitherto unearthed although few reports are there for its wide range of biotechnological applications [19], [20]. Targeting the hypervariable V3 region, a total of 22.44 million paired end raw reads were obtained for five marine samples using Illumina GAIIx platform. Averages of 4.48 million reads were obtained for each sample. Stringent measures were taken to rule out the erroneous reads. Raw reads with non ATGC characters, primer containing sequences and possible adapter contaminated sequences were trimmed and taken for subsequent analysis. In order to eliminate the low quality reads, reads with more than 70% of bases with Phred value of greater than 20 alone were taken into account. Since chimeric reads tend to inflate bacterial diversity, they were removed using CD-HIT DUP and nearly 3.93 million chimeric pruned reads were obtained from all the five marine samples [21]. These stringent measures removed nearly 87% of the initial reads which is in accordance with other reports that filter 50–80% of initial reads [8], [9], [22] (Table 1).

**Table 1:** Overview of sequence information.

Sample	Barcode	No. of raw reads	No. of high quality reads^a^	No. of reads that passed CD-HIT DUP^b^	No. of reads with predicted rRNA like sequences	No. of reads with identified rRNA sequences
		(in millions)				
R-SED	CGTACT	4.24	3.79	1.10	0.54	94151
M-SED	CTGATC	4.78	4.37	0.54	0.29	71112
SR	CACTGT	3.20	2.84	0.78	0.38	46245
SW	GACTGA	5.12	3.60	0.69	0.34	68503
SG	CGTACT	5.10	4.63	0.82	0.42	80316

a More than 70% of bases in a read with >20 phred score and reads which are of low quality were trimmed and used.

b Number of reads after chimera and duplicate removal.

### Diversity Index and Taxonomic assignment at Phylum level

**Figure 1:**
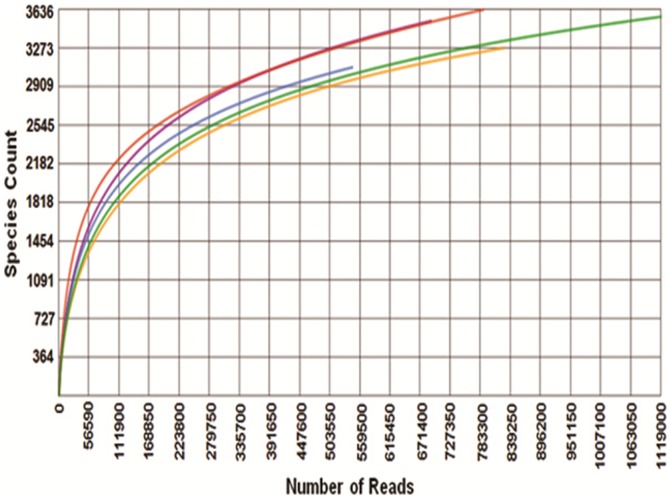
Rarefaction analysis for the observed species. The rarefaction curves for all the marine samples reached the near plateau phase representing good sampling depth. The samples are R-SED (Green), M-SED (Blue), SR (Red), SW (Violet) and SG (Orange).

**Figure 2:**
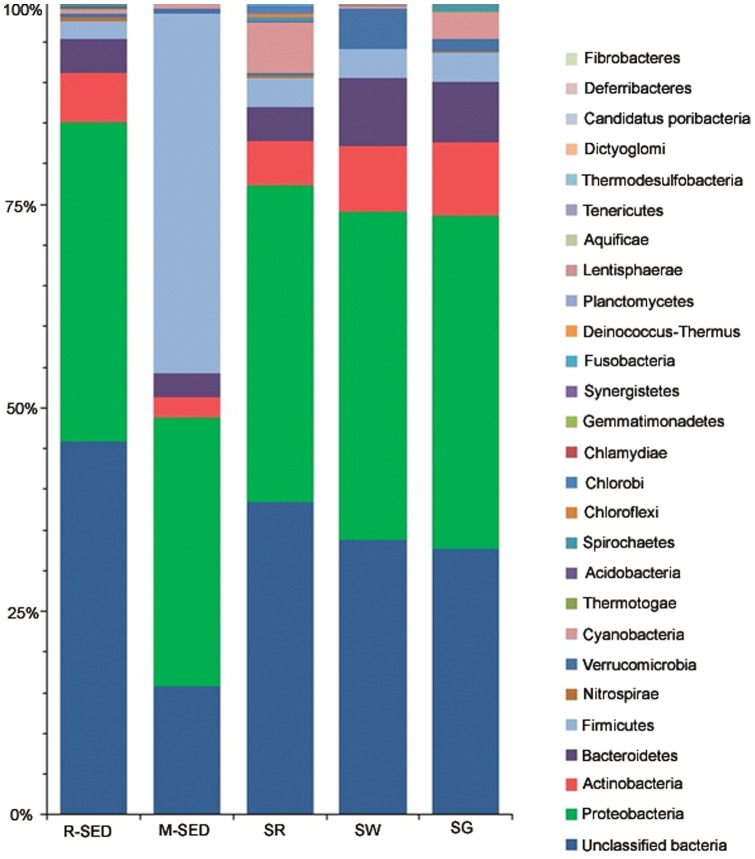
Relative abundance of bacterial phyla across the samples. The percentage of sequences is plotted on Y-axis. Unclassified bacteria were reads without recognizable match in the searched database. Except for M-SED, Proteobacteria is the predominant phylum in all the samples. In M-SED, Firmicutes is the predominant phylum.

**Figure 3:**
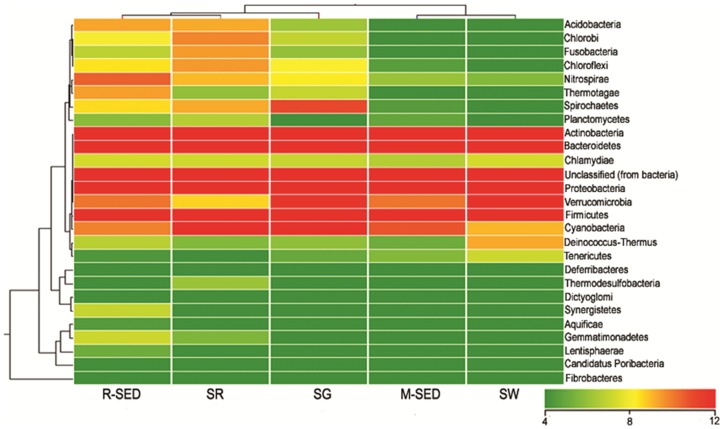
Heat map analysis of the marine samples. The marine samples do not cluster together indicating that the bacterial diversity among the samples is varied and distinct.

Taxonomy assignment was done using MG-RAST pipeline [23]. For all five samples the rarefaction curves have reached near plateau, indicating that the sampling depth and sequencing coverage were good (Figure 1). Both the Shannon weiner index and Simpson index showed that marine sediment had the highest diversity while rhizosphere sediment had the lowest (Table 2). The relative distribution of each phylum varied among the marine samples. The predominant phyla observed in all the five marine samples were Proteobacteria, Actinobacteria and Bacteroidetes. This result supports the fact that the representative members from these phyla are cosmopolitan and are not specific to the specific habitat. This is the first report that documents the presence of different bacterial phyla on the surface of seaweed and seagrass. The relative abundance of each phylum varied among rhizosphere sediment, marine sediment, seawater, seagrass and seaweed and remains distinct to each other (Figure 2). This result was also complemented by the heat map analysis in which these marine samples do not cluster together (Figure 3). The relative abundance of firmicutes (*Bacillus* and *Halobacillus*) was higher in sediment sample and rather it was lower in other marine samples. This report is in total agreement with earlier reports wherein the higher abundance of firmicutes in sediment is attributed to human fecal contamination [24], [25]. In addition, other possible reasons for the abundance could be the ability of *Bacillus* sp to form endospore [26]; Firmicutes are better competitors in the natural environment since they are widely recognized as producers of enzyme inhibitors, antibiotics and its metabolic and physiologic versatility [27]. Furthermore, seawater contains high proportion of cyanobacteria which was almost rare in other marine samples. One may hypothesize that since most of the cyanobacteria are photosynthetic, it is freely floating in seawater and it may not carry out photosynthesis effectively if it is associated with other marine samples. In general, the relative abundance of each phylum among the marine samples was highly variable.

**Table 2:** Diversity indices of the marine samples.

Sample	Diversity Index	
	Shannon Index	Simpson Index
R-SED	3.49	0.20
M-SED	4.30	0.04
SR	3.63	0.15
SW	3.75	0.12
SG	4.10	0.10

### Distribution of bacterial communities at the higher taxonomic level

**Figure 4:**
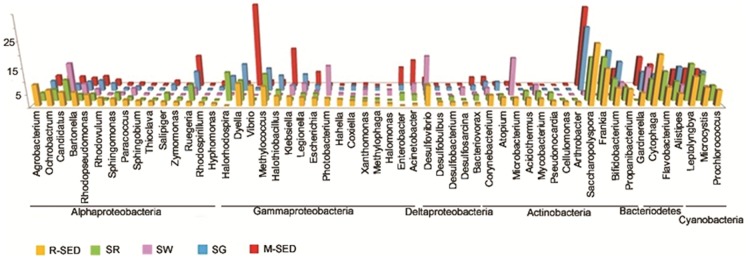
Comparison of bacterial communities among the samples at genus level. The members of the phyla Alphaproteobacteria, Gammaproteobacteria, Deltaproteobacteria, Actinobacteria, Bacteriodetes and Cyanobacteria which are above 1% are compared among the samples. As evident all the samples had varying levels of different genera.

We assessed the relative distribution of bacterial taxa at the level of class and genus for each sample. Except seawater, Alphaproteobacteria was abundant in all other marine samples. Though the high abundance of Alphaproteobacteria has been well documented in seawater, the relative low abundance of this class in seawater in the present study may be explained by difference in various ecophysiological factors, which is not the focus of the present study. The representative members of genera *Agrobacterium, Ochrobactrum, Bartonella, and Ruegeria* were most often present in all the marine samples. Nearly equal distribution of Gammaproteobacteria was observed in rhizosphere sediment, seawater, seaweed and seagrass samples whereas increased abundance was observed in sediment sample. The dominant genera of Gammaproteobacteria like *Halorhodospira, Dyella, Vibrio, Methylococcus, Halothiobacillus, Klebsiella, E.coli, Enterobacter and Acinetobacter* were present in all the marine samples in various levels. This observation is in parallel with the other global studies in which Gammaproteobacteria was found to be widely distributed in different marine niches supported by its huge phylogenetic and phenotypic diversity [28]. Several intermediate abundant taxa like Actinobacteria, Flavobacteria and Optitutae were present in all the marine samples. *Desulfovibrio*, the most dominant genus from Delta proteobacteria was found to be abundant in seaweed. The other members of the class like *Desulfovibrio, Desulfobulbus, Desulfosarcina, Sulfobacterium and Bacteriovorax* were scarce in all the samples. Several reports documented the presence of members of Deltaproteobacteria in marine environment which mainly acts as sulfate reducers [29] (Figure 4).

At phylum level, most of the bacterial taxon was recurrently found in all the marine samples, but at higher taxonomic level (genus and species level) the relative contribution of the bacterial taxa of the community varied from one sample to other. This suggests that though all the samples are geographically linked to each other, the composition of bacterial taxon at the genus and species level gets varied among each other. The reasons for this characterization include the fact that each marine sample provides different nutrient composition and varied ecological conditions for specific bacterial taxon to reside and proliferate. Further analysis was carried out to understand how these marine samples are distinct to each other in terms of both abundant and rare species diversity. The details of taxonomic affiliation for all the samples have been provided in Table S1 to S5. These tables provide the detailed view of taxonomic level distribution of sequences from phylum to strain level and their corresponding number of hits in descending order of their relative abundance (%). The hits are based on clustering the sequences at 97% identity against RNA databases implemented in MG- RAST (namely RDP, Greengenes, SILVA LSU and SSU). Graphical representation of the relative abundance of bacterial diversity from phylum to species can be visualized using Krona chart in Figure S1 to S5.

### Analysis of abundant and rare species diversity in marine samples

**Figure 5:**
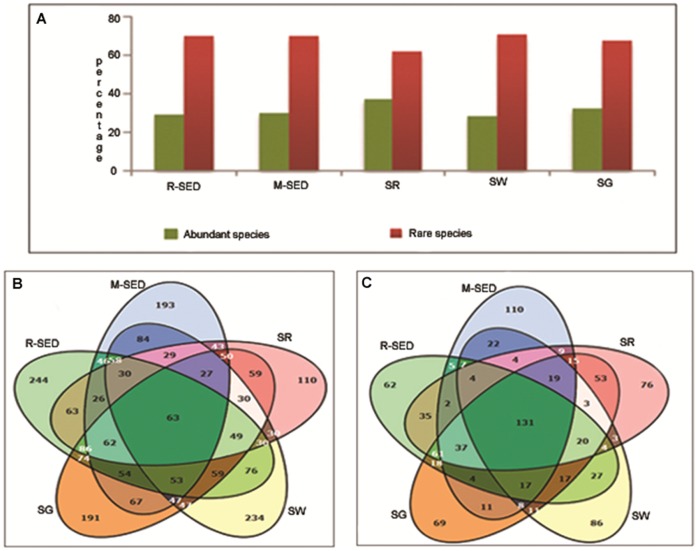
Analysis of abundant and rare species among the marine samples. A. **Percentage of abundant and rare species across the samples.** In all the five samples when compared to abundant species the percentage of rare species is significantly high indicating the importance of rare species in a community. B. **Shared and specific rare species among the marine samples.** Most of the rare species are unique to particular sample and only a few are shared by all the samples. C. **Shared and specific abundant species among the marine samples.** Most of the abundant species are common to all the samples and only a few are unique to particular sample.

Both abundant and rare species constitute the bacterial population in all the marine samples. Based on the previous reports [11], [30] the rare species may be defined as the frequency of species with <0.01% of the total population and the rest of the population are considered to be abundant. According to this the proportion of abundant and rare species ranges from 28.12% to 37.7% and 62.3% to 71.18% respectively in all the marine samples (Figure 5A). Venn diagram was constructed to predict the number of unique and shared species among the abundant and rare species in five marine samples. It was observed that few of the rare species were shared commonly among the samples (Figure 5B) and most of the species which were unique to a particular sample are rare (Figure S6). But in case of abundant species, most of the abundant species were shared among the samples and only few are unique (Figure 5C). Details of the unique and rare species corresponding to each marine sample are given in Table S6. It has been a long belief that abundant species that is represented by few species in the bacterial population plays an important role in marine habitat. But the present report provides a testament that rare species also play an important role in biogeochemical cycle and other functional role in the marine environment. In addition, the rare species which are unique to a particular sample may play important roles than previously predicted. Further research regarding the importance of rare species in the marine environment is needed to fully understand its role in the marine ecosystem.

In summary, we report that, though these marine samples are geographically linked to each other, the bacterial community compositions are distinct to each other at higher taxonomic level. This suggested that, distribution of bacterial community composition was very specific to specific habitat even though these habitats are present in similar geographical location. In addition, though high throughput sequencing using illumina depicts the known bacterial community of marine samples in the present study, the unclassified bacteria constitute a wide range of 15% to 45% for all the marine samples. Further studies like whole metagenome sequencing holds promise to delineate the uncultured bacterial diversity of these samples. Moreover, this is the first study that sheds light on hitherto unexplored bacterial diversity of geographically similar marine samples in Palk Bay region which hold greater promise for broad range of biotechnological applications.

## Materials and Methods

### Ethics Statement

No specific permissions were required for the present study described here. The location is neither privately-owned nor protected in any way and this study did not involve any endangered or protected species.

### Sample collection

Marine sediment (M-SED), rhizosphere sediment (R-SED) (*Rhizophora apiculata*), sea water (SR), seaweed (SW) (*Gracilaria* sp.) and seagrass (SG) (*Cymodacea* sp.) were collected from Karankadu Coastal area (8° 28′N lat. and 77° 41′E long.), Palk Bay, Tamil Nadu, India. The sampled seaweed and sea grass are the most predominant ones in the sampling site. All samples except seawater were collected in sterile plastic bags. Seawater was collected in sterile can and initially filtered to remove the debris followed by subsequent filtration with 0.22 micron filter (Millipore, Massachusetts, US). All the other samples were transported to laboratory within two hours in ice and later stored at −80°C until processing.

### DNA extraction

DNA from sediment and rhizosphere sediment were extracted according to the lyzozyme method as described [31]. DNA extraction from filters was done using standard procedure [13]. DNA extraction from the surface of seaweed and sea grass were done as previously described [32]. Extracted DNA was visualized by agarose gel electrophoresis and quantified using nanodrop spectrophotometer (Shimadzu, Singapore).

### Amplicon library construction

Library construction involved two PCR reactions (Kapa HiFi Hot start, Kapa Biosystems, Massachusetts, US). The first reaction targeted the V3 region using primers 341F, 5′-CCTACGGGAGGCAGCAG-3′ and 518R, 5′-ATTACCGCGGCTGCTGG-3′ with the initial amount of 20 ng of DNA. Cycle conditions were an initial denaturation at 98°C for 3min, followed by 20 cycles at 98°C for 30 sec, 60°C for 30 sec, 72°C for 30 sec, and ended with a final extension step at 72°C for 3 min. The amplified products of 170–200 bp were purified by gel elution using minelute columns (Qiagen, Hilden, Germany). For the subsequent cycle of PCR, 150 ng of the amplified sample was used as template and modified primers (which included V3 specific sequences and adapter sequences) were used as suggested [33]. These modified primers had four degenerate bases in the forward primer to enhance base calling accuracy, cluster density and aids in identifying unique clusters. The reverse primer contains a 6-bp barcode sequence for multiplexing. Similar PCR conditions were used as the initial protocol except the number of cycles were reduced to six. The amplified products were cleaned up using Agencourt Ampure XP SPRI beads (Beckman Coulter, California, US). The prepared library was validated for its quality by running an aliquot through High Sensitivity Bioanalyzer Chip (Agilent technologies, California, US) and quantified using nanodrop spectrophotometer.

### Illumina sequencing

PCR products with unique indices from each library were taken in equal nanogram quantities and subjected to 100- nucleotide paired-end multiplex sequencing using Illumina GAIIx sequencer at Genotypic Technology Pvt. Ltd (Bangalore, India). All the samples except R-SED were pooled in a single lane. R-SED amplicons were sequenced in another lane combined with other samples not related to this work. Image analysis and base calling were done using Illumina Analysis pipeline (Version 2.2).

### Initial Processing of sequence reads

Demultiplexing was done using Consensus Assessment of Sequence and Variation (CASAVA). Only high quality reads with more than 70% of bases with Phred Score greater than 20 were considered significant and taken for subsequent analysis. Reads with adapter sequences were removed. The primer sequences, the barcode and the degenerate bases were removed using an automated Perl code generating processed reads.

### Data analysis

Duplicates and chimeras from processed reads were removed using CD-HIT DUP with the minimum length of common sequence shared between a chimeric read and each of its parents set as 20 bases [34]. Abundance ratio between a parent read and a chimeric read was set to 1. The resulting dataset was pre-screened using uclust for a minimum of 70% identity to ribosomal sequences and then clustered at 97% identity against RNA databases implemented in MG- RAST (namely RDP, Greengenes, SILVA LSU and SSU) [35]. Taxonomic assignment from phylum level to strain level was assigned based on the hits. Abundance graphs were plotted based on the number of hits. Heatmap and rarefaction curve were plotted using MG-RAST. The Venn diagram was made with Venn diagram plotter jquery.venny, a tool developed by genotoul bioinfo (http://bioinfor.genotoul.fr). Diversity index was calculated using SPADE software [36]. Krona graphs were plotted using Krona [37].

### Nucleotide sequence accession number

Paired end Illumina sequence data from this study were submitted to the NCBI Sequence Read Archive (SRA) under accession number SRP018711.

## Supporting Information

Figure S1 **Graphical representation of the relative abundance of bacterial diversity from phylum to species level of M-SED can be visualized in this file using Krona visualization tool.** This file will help the readers to understand the relative distribution and abundance of the complex bacterial community composition of the sample through simple graphical representation.
(HTML)

Figure S2 **Graphical representation of the relative abundance of bacterial diversity from phylum to species level of R-SED can be visualized in this file using Krona visualization tool.**
(HTML)

Figure S3 **Graphical representation of the relative abundance of bacterial diversity from phylum to species level of SR can be visualized in this file using Krona visualization tool.**
(HTML)

Figure S4 **Graphical representation of the relative abundance of bacterial diversity from phylum to species level of SW can be visualized in this file using Krona visualization tool.**
(HTML)

Figure S5 **Graphical representation of the relative abundance of bacterial diversity from phylum to species level of SG can be visualized in this file using Krona visualization tool.**
(HTML)

Figure S6 **This file is a bar graph describing the percentage of abundant and rare species among the unique species of the five marine samples.**
(TIF)

Table S1 **This file contains the details of taxonomic level distribution for M-SED sample.** Details of phylum to strain level distribution and their corresponding number of hits are given by clustering the sequences at 97% identity against RNA databases implemented in MG- RAST (namely RDP, Greengenes, SILVA LSU and SSU).
(XLSX)

Table S2 **This file contains the details of taxonomic level distribution for R-SED sample.**
(XLSX)

Table S3 **This file contains the details of taxonomic level distribution for SR sample.**
(XLSX)

Table S4 **This file contains the details of taxonomic level distribution for SW sample.**
(XLSX)

Table S5 **This file contains the details of taxonomic level distribution for SG sample.**
(XLSX)

Table S6 **This file contains the details of shared and specific abundant species among the five samples in the first sheet and the details of shared and specific rare species among the samples in the second sheet.**
(XLS)
