# Supplementary material for: Ultradeep 16S rRNA Sequencing Analysis of Geographically Similar but Diverse Unexplored Marine Samples Reveal Varied Bacterial Community Composition
Source: PLoS One. 2013 Oct 22;8(10):e76724. doi: 10.1371/journal.pone.0076724 (PMC3805540; doi:10.1371/journal.pone.0076724)
Supplement: Figure S1 — Graphical representation of the relative abundance of bacterial diversity from phylum to species level of M-SED can be visualized in this file using Krona visualization tool. This file will help the readers to understand the relative distribution and abundance of the complex bacterial community composition of the sample through simple graphical representation. (HTML) [file pone.0076724.s001.html]

Javascript must be enabled to view this page.

members
magnitude

Repl\_SED\_krono

199439

0

0

0

0

0

0

0

199439

6015

294

294

257

83

1

2

4

2

74

59

59

115

81

34

28

23

23

5

5

9

9

9

973

973

950

852

852

98

98

10

10

10

4

4

4

2

2

2

7

6

1

1

3

1

1

1

1649

1649

796

1

1

2

2

5

1

3

1

7

7

1

1

10

10

708

1

56

236

21

11

6

2

26

63

2

192

6

71

14

1

62

62

284

284

284

569

563

563

6

6

220

220

220

13

13

95

65

30

3

3

89

89

20

20

2879

78

78

78

11

67

2801

2801

46

46

143

143

26

26

104

1

103

2

2

21

19

2

20

1

19

345

73

4

268

517

517

517

122

67

11

110

6

2

130

65

4

2

2

188

188

48

1

47

588

250

17

293

10

18

8

8

19

19

2

2

53

53

8

8

18

18

9

9

103

103

14

14

99

99

99

2

2

2

66

2

2

18

18

46

36

10

1

1

1

30

18

18

12

12

1700

1691

1

1

1

1

192

192

20

19

1

108

100

8

6

3

3

11

4

3

4

5

5

2

2

8

7

1

29

20

9

3

3

113

1

1

1

112

112

112

8

8

4

4

2

2

2

2

57

33

3

3

9

9

10

1

7

2

5

5

2

2

1

1

3

1

2

24

24

21

3

1320

1320

2

2

1

1

1190

134

109

3

24

162

1

83

4

472

3

9

26

1

2

9

91

23

1

33

2

2

115

54

2

16

43

4

4

6

1

3

2

9

9

9

9

9

48

48

35

35

35

12

4

19

10

10

10

1

2

2

1

1

1

1

1

3

3

3

1

2

16

16

16

16

6

5

1

10

10

12

12

12

12

12

12

10

10

10

10

4

4

1

1

5

2

1

1

1

67

67

67

67

1

1

21

21

20

20

25

1

24

4762

4762

7

7

7

7

4269

41

1

1

2

2

1

1

6

6

31

30

1

1

1

1

35

35

6

29

28

28

12

1

10

5

15

15

15

85

85

85

411

411

411

3

3

3

139

139

2

136

1

1

1

1

35

2

2

33

33

25

9

5

4

16

16

620

4

4

1

1

615

615

1

1

1

163

163

1

1

2

1

7

1

5

1

4

6

3

122

8

1

28

22

5

3

2

3

1

3

5

6

6

5

5

5

230

68

68

2

2

4

3

1

9

9

62

2

11

2

9

14

23

1

29

29

1

1

40

1

1

38

15

15

1

1

1

1501

7

2

3

2

1494

778

290

408

18

10

10

10

54

21

21

33

33

215

160

160

54

54

1

1

53

53

21

23

9

336

14

14

3

3

319

70

2

243

4

222

222

10

212

2

2

1

1

9

9

9

400

400

390

45

6

111

171

2

4

13

23

1

2

4

8

10

10

83

83

2

2

55

18

34

3

8

8

18

18

3

3

2

2

1

1

31452

31452

31452

31452

31452

25

30525

41

33

116

2

1

63

7

84

1

2

14

20

1

10

507

66095

144

26

26

2

2

1

1

23

15

6

1

1

118

118

1

1

112

112

5

5

19359

301

162

72

1

71

30

28

2

37

37

2

2

7

2

3

2

14

14

139

18

18

2

2

8

8

111

16

24

71

6943

6943

5

5

6937

4

6933

1

1

458

444

3

3

119

119

15

1

14

125

3

122

182

3

2

177

14

2

2

12

6

5

1

5409

602

602

137

3

3

459

219

219

219

792

792

767

11

14

124

12

12

101

96

3

1

1

11

1

8

2

1

1

1

453

83

83

339

1

18

4

5

1

143

11

7

5

110

34

29

29

2

2

271

270

270

1

1

123

19

19

104

104

76

76

76

1570

42

3

3

15

3

18

22

4

18

11

11

709

709

786

786

268

268

99

168

1

786

1

1

4

4

744

744

37

1

18

18

124

124

124

33

33

2

2

1

1

30

13

4

13

92

2

2

2

9

9

9

35

5

5

2

2

28

28

46

19

6

13

8

7

1

7

7

12

7

5

3

3

3

3

6120

360

31

31

3

3

293

292

1

33

33

1

1

1

5759

923

923

15

2

9

4

18

18

119

119

347

34

149

38

2

6

112

6

88

87

1

3

3

1

1

4

4

2338

1483

91

4

13

747

15

15

529

21

5

424

76

3

214

12

19

11

169

3

178

177

1

1

1

1

1

90

90

378

378

5

5

27

27

5

5

455

188

35

3

208

21

5

5

38141

68

68

1

1

11

11

1

1

29

29

26

25

1

65

65

59

17

3

1

3

3

6

26

6

6

51

51

49

49

2

2

768

768

1

1

4

4

1

1

2

2

760

760

485

5

5

1

4

46

46

1

25

5

15

12

12

12

238

238

1

20

153

53

1

10

6

6

6

178

163

10

3

149

1

3

3

3

3

8

8

1

1

746

503

503

436

45

1

21

243

220

217

3

23

23

12753

12753

270

26

1

181

11

3

38

10

7

7

10

10

1

1

12341

95

223

40

6

740

51

646

1

14

16

11

205

27

2

3

4

295

1

1

2

1

44

49

8

5

18

34

1

1

1

20

8

3

4

2

31

47

6

1

48

3

51

108

7

7792

14

27

4

24

1572

18

5

1

124

124

2952

2952

16

16

2934

1

5

88

1

145

4

2

9

2531

47

1

2

78

12

8

2

2

971

971

272

1

13

252

1

5

131

123

8

568

568

89

25

25

2

3

1

12

1

6

6

6

6

58

58

58

1229

307

307

307

132

7

7

1

1

90

90

20

20

1

1

1

1

10

9

1

2

2

790

790

546

244

12942

12942

14

14

5

5

793

2

787

2

2

2

2

5692

20

5663

6

3

2819

1178

1

1578

18

2

19

20

3

92

88

4

29

29

3

3

74

74

53

53

9

9

34

34

105

72

1

32

44

2

42

155

4

135

7

9

4

4

699

3

221

191

11

259

14

8

8

2103

2

2015

59

27

73

1

69

2

1

83

12

32

39

45

45

2

1

1

2

2

308

123

123

123

16

2

2

14

1

6

7

57

56

5

4

47

1

1

71

71

71

41

41

41

4714

789

16

16

773

18

351

7

1

129

10

161

47

3

1

33

3

1

2

6

3925

3

3

18

16

1

1

3904

47

1

51

515

2809

3

34

237

30

3

22

45

107

2653

1467

143

15

2

1

12

117

19

96

2

11

5

3

3

110

76

1

1

29

34

4

7

26

1

18

1

2

4

8

1

7

27

7

1

6

1

1

1

1

9

9

9

9

1139

28

28

360

360

751

751

48

1

1

3

3

1

1

4

4

2

1

1

25

3

11

1

3

6

1

4

2

2

7

3

4

1

1

207

207

53

53

154

102

52

886

886

33

33

1

1

120

120

615

381

234

53

2

51

64

24

1

4

24

4

2

5

87

87

77

10

67

10

10

6

4

4

4

2

2

2

4772

3900

3900

3868

3791

5

2

2

68

32

32

56

4

4

4

9

3

3

6

6

10

3

3

7

7

2

2

2

8

8

8

23

6

6

17

17

235

10

10

10

37

6

4

2

31

31

147

147

25

17

29

5

3

21

1

1

30

3

1

3

7

1

41

41

41

76

22

4

4

18

8

10

14

14

14

6

5

5

1

1

34

34

34

338

33

2

2

2

2

1

1

23

23

5

5

305

13

1

12

18

6

12

15

15

157

145

12

15

8

7

13

13

11

11

63

63

160

30

30

1

8

1

20

130

130

130

7

7

2

2

5

5

1026

1026

1026

1022

126

1

895

4

4

29

29

29

29

19

3

13

3

8

8

2

2

23

23

23

23

3

3

11

5

1

3

2

9

2

7

29

1

1

1

1

1

14

14

14

14

14

14

14

14

2

2

12

5

2

5

87812

1074

128

66

4

4

2

1

1

55

55

1

1

2

2

2

2

62

62

54

2

6

7

7

5

5

2

1

1

1

1

1

1

938

114

75

75

6

6

5

5

5

5

23

2

5

2

14

1

1

1

7

5

5

1

1

1

1

8

4

3

1

2

1

1

2

1

1

60

60

60

43

1

1

33

1

8

2

6

4

4

1

7

9

9

470

12

12

447

66

2

3

2

4

8

8

2

14

2

1

2

21

14

1

1

1

1

2

2

6

1

1

42

2

1

4

21

3

15

9

9

3

9

7

56

48

27

3

13

3

7

11

5

6

24

16

16

1

1

7

7

97

1

1

68

68

11

1

10

3

1

2

14

1

13

1

1

1

113

18

14

4

1

1

2

2

91

17

1

12

4

14

1

11

15

1

5

8

2

1

1

84331

1795

30

18

10

4

2

1

1

12

11

1

13

1

1

2

2

10

5

5

1395

229

2

227

56

3

53

1110

142

953

15

8

7

1

2

1

3

1

1

317

236

3

3

1

50

14

4

1

4

4

6

1

70

1

1

1

10

2

6

3

1

2

1

2

2

43

81

1

26

34

20

32

13

13

19

14

5

82536

463

29

29

434

46

19

48

18

10

6

1

1

1

17

2

1

3

25

1

1

3

219

4

8

24

9

9

15

1

14

36

10

2

1

7

14

14

6

5

1

6

6

319

187

9

16

7

17

8

75

1

5

1

3

1

13

5

5

1

1

19

46

40

6

86

4

7

1

19

3

5

47

81455

775

61

136

3

24

550

1

117

33

84

23

17

6

16

14

2

303

281

12

6

1

3

181

13

54

38

21

1

1

2

1

5

36

9

28

22

6

58475

9

566

100

6

70

532

10

610

45

73

87

544

905

38

686

1315

4922

157

2

23

2

11150

1

2154

1

1094

12

1

1

1

10

6

12

2797

21

1

207

21

1489

382

84

7472

2355

90

8147

5

1474

14

1

2

1443

11

92

38

2

5

1

18

32

1845

19

77

2

2

58

806

3

82

3

4

20

523

125

3

2165

892

4

10

25

2

39

57

239

121

21460

10

1215

19

19202

66

55

53

347

16

6

165

306

3

3

73

25

48

1

1

54

50

6

14

5

1

2

21

1

4

4

4

4

4

160

2

2

158

158

21

3

3

3

2

1

15

15

2381

2381

1042

1042

803

239

1339

3

3

344

277

67

1

1

188

188

25

25

3

3

765

15

750

2

1

1

8

8

26

26

26

5

5

14

14

7

7

35

35

6

6

6

6

29

29

29

24

4

1

1223

55

54

54

54

54

1

1

1

1

103

101

101

101

101

2

2

2

2

1065

1065

2

2

2

1063

322

1

321

35

35

101

101

1

1

604

2

250

352

12

12

12

12

5

5

7

2

5
