# Supplementary material for: Ultradeep 16S rRNA Sequencing Analysis of Geographically Similar but Diverse Unexplored Marine Samples Reveal Varied Bacterial Community Composition
Source: PLoS One. 2013 Oct 22;8(10):e76724. doi: 10.1371/journal.pone.0076724 (PMC3805540; doi:10.1371/journal.pone.0076724)
Supplement: Figure S2 — Graphical representation of the relative abundance of bacterial diversity from phylum to species level of R-SED can be visualized in this file using Krona visualization tool. (HTML) [file pone.0076724.s002.html]

Javascript must be enabled to view this page.

members
magnitude

R\_SED\_krona

266103

0

0

0

0

0

0

0

266103

8

8

8

8

8

8

11311

635

635

270

61

4

24

33

86

86

123

1

4

90

1

1

26

20

7

7

13

4

9

342

342

342

3

3

3

895

895

774

236

236

538

394

144

89

89

89

1

1

1

22

22

4

3

15

9

9

1

1

1

3

3

5092

5092

2808

6

6

7

7

64

5

1

3

1

54

97

97

12

12

23

5

18

2295

26

6

32

231

315

162

82

3

43

1

381

1

117

82

667

24

122

304

304

1030

1030

1030

1254

1234

1234

20

20

648

648

648

14

14

202

145

57

117

117

293

293

22

22

4041

249

249

249

1

5

243

3792

3792

78

78

198

198

6

1

5

187

6

6

175

3

3

313

244

69

19

3

16

493

133

20

340

461

461

815

236

70

48

81

46

2

240

81

11

1

1

117

117

9

2

7

222

26

3

112

5

76

43

43

8

5

3

5

3

2

421

421

34

34

47

47

15

15

29

29

111

111

157

157

158

158

158

119

31

31

25

25

63

39

24

13

13

13

26

1

1

6

6

19

19

1035

1019

6

6

2

2

4

4

144

144

8

8

108

1

104

3

4

3

1

2

1

1

7

1

2

2

2

1

1

3

1

2

2

2

2

2

5

5

2

2

62

3

3

3

59

59

59

2

2

2

2

5

5

3

3

2

2

40

32

4

4

3

2

1

4

4

7

7

3

1

1

1

11

11

2

2

2

6

6

6

760

760

5

5

8

8

1

1

68

37

2

4

25

19

1

2

16

651

123

1

123

2

1

59

6

1

1

77

124

2

87

2

27

1

14

7

7

1

1

16

16

16

16

16

20

20

13

13

13

1

7

1

1

2

1

7

7

6

5

1

1

1

683

683

683

683

25

25

8

3

5

1

1

159

134

8

1

16

490

485

5

632

310

310

310

3

3

1

1

306

306

25

25

25

25

25

297

297

297

297

297

112

112

112

112

14

14

27

27

38

36

2

33

3

9

8

13

138

138

138

138

138

138

1415

1415

1415

1415

12

2

10

436

436

882

882

85

6

67

12

5

5

5

5

2

2

2

2

1

1

16105

16105

64

64

64

64

13100

5

5

5

98

98

45

53

44

20

19

1

22

1

4

3

14

2

2

1

1

1

490

490

490

85

53

53

32

32

253

6

2

1

3

33

6

27

5

5

1

1

12

12

196

196

1

1

1

669

669

1

3

17

3

1

8

1

1

9

3

6

1

3

41

1

1

7

1

1

1

15

4

1

1

224

99

215

100

66

15

6

2

3

19

6

2

2

11

34

32

1

1

987

19

19

1

1

165

23

135

7

37

37

55

55

65

65

3

3

503

2

1

1

51

97

1

1

1

1

1

156

183

3

4

139

139

1

1

1

4167

311

260

11

10

1

3

2

1

23

6

5

1

6

6

3837

1412

497

1571

7

350

6

6

1

1

180

129

129

47

25

22

4

3

1

183

22

21

1

161

43

117

1

78

78

2

2

72

2

3

3

3

2

1

1

1

1

284

31

30

1

4

4

16

2

5

9

233

172

1

28

1

4

27

485

485

66

2

32

37

1

5

16

3

70

1

25

1

1

37

158

1

1

5

3

3

3

1

1

3

8

1

2307

2307

2307

178

175

175

3

3

278

2

1

1

276

104

5

152

14

1

155

136

2

134

19

19

366

264

39

225

102

102

8

8

5

3

52

52

7

2

7

36

5

5

2

3

8

8

8

34

8

8

1

1

25

25

1346

73

73

233

233

1040

70

11

903

56

76

1

1

75

1

2

1

1

1

4

3

2

12

3

1

36

1

6

1

164

164

164

7

7

7

4

4

4

4

5

5

5

5

1931

4

4

4

1927

1114

2

157

90

17

3

14

20

75

138

2

318

12

40

1

1

224

813

813

838

838

59

31

28

8

1

7

527

293

234

130

130

2

2

106

106

6

6

163

163

7

7

156

156

22

22

22

22

17

17

5

5

121887

121887

121887

121887

121887

23

118245

38

1

75

145

563

152

3

445

26

57

4

3

59

39

286

1723

104378

1266

12

12

9

9

3

3

384

4

4

4

380

20

4

16

7

7

353

233

95

4

14

7

870

870

34

2

32

836

836

42078

2951

1548

742

742

14

14

390

390

37

37

151

2

136

5

8

214

28

186

1403

12

12

2

2

524

9

24

205

286

74

74

70

1

64

2

1

1

1

1

1

700

700

16

16

4

4

12364

12364

103

103

12256

2

1

52

52

12149

5

5

2822

2785

15

15

630

630

118

9

42

16

51

846

846

1176

5

29

2

1

1117

22

37

16

16

21

2

4

3

9

3

16068

2212

2212

444

16

106

1646

450

450

449

1

2597

2596

2

10

1735

841

7

1

1

1

1147

60

17

36

7

958

907

11

26

1

1

12

129

16

75

22

7

8

1

18

18

18

377

13

13

224

3

2

12

14

2

1

82

1

5

4

61

37

116

113

3

24

24

110

28

28

64

1

63

14

14

4

4

634

2

2

5

5

315

315

312

312

43

43

43

5900

106

4

2

3

15

82

75

8

67

37

37

2231

2231

3451

3451

343

343

290

52

1

1964

22

22

1827

1809

18

115

23

40

52

273

273

273

26

26

3

3

23

8

15

241

21

3

3

18

18

23

23

23

29

3

3

26

26

168

90

23

67

18

18

1

1

55

7

46

1

1

4

4

66

66

66

66

7540

735

202

202

3

3

413

413

117

117

4

4

4

6801

315

315

49

49

182

4

8

144

11

4

11

272

272

377

1

211

54

7

21

83

8

6

2

29

28

1

22

22

2

2

13

13

602

360

36

1

1

1

203

53

53

737

1

12

702

10

12

16

16

988

6

24

6

2

7

1

31

891

20

373

362

11

6

6

731

731

1

1

681

681

3

3

20

20

2

2

3

3

1285

486

123

2

528

2

2

4

138

7

7

11

11

13

10

3

30716

123

123

2

2

10

10

8

8

2

2

5

5

12

11

1

2

2

82

63

2

1

16

51

51

9

1

4

4

42

42

21

21

21

21

2401

2401

2

2

11

11

15

15

1

1

51

51

2321

2321

739

133

133

4

2

117

2

8

6

6

6

65

65

65

245

245

1

1

8

129

1

3

5

93

4

9

9

7

2

281

2

1

1

100

16

1

35

48

41

8

13

1

5

14

10

5

2

3

56

56

11

11

60

51

8

1

1

1

1665

1006

1006

989

1

6

1

9

659

636

610

24

2

23

23

3595

3595

910

3

855

8

1

4

2

18

18

1

114

1

113

3

3

2443

19

61

1

60

44

1

46

23

16

74

43

1

2

38

1

1

8

44

7

9

1

1

10

38

19

22

8

30

1

6

2

2

4

276

6

1

8

2

4

3

3

9

21

618

1

29

1

1

5

35

187

37

374

20

153

3

3

1

1

124

124

6

6

6

6

10299

10299

12

12

7

7

13

12

1

2

2

106

106

10155

8

7

151

1

16

11

59

47

130

133

74

134

114

29

9087

71

83

4

4

3256

3256

560

2

12

1

525

16

4

22

22

2674

2674

509

54

54

9

1

6

1

2

5

30

50

50

41

2

7

405

15

15

30

30

7

7

353

345

8

3639

1585

1483

1483

1

1

12

12

3

1

2

13

1

6

6

73

1

71

1

893

8

8

8

8

5

1

4

1

1

589

2

587

4

4

7

4

3

60

60

14

14

159

1

125

4

26

3

3

3

3

3

3

3

27

27

2

2

1161

1161

656

436

4

63

2

2537

2537

1

1

4

4

13

10

1

2

24

24

1112

2

1110

6

5

1

2

2

21

21

30

16

14

23

4

2

17

7

7

2

2

921

780

141

340

17

6

1

255

61

14

14

14

14

1

1

1

1

1

1

1398

801

801

801

286

4

4

282

1

62

219

111

8

8

1

1

97

84

3

10

1

1

4

1

3

166

166

4

34

15

6

18

89

34

34

34

477

269

38

38

231

14

133

1

13

6

3

1

29

21

3

7

208

1

1

207

3

2

2

116

17

62

5

2403

2

2

2

2

952

115

114

2

112

1

1

483

474

243

26

6

191

8

9

7

1

1

36

26

26

5

5

2

2

3

3

224

35

35

188

188

1

1

94

6

6

18

18

1

1

18

18

7

2

5

14

4

10

12

1

2

1

8

3

3

15

3

12

1118

1118

4

4

159

20

119

20

955

292

663

61

61

47

47

10

10

2

2

2

1

1

132

132

127

127

5

5

129

117

117

117

12

1

1

5

5

6

3

2

1

9

9

9

9

24887

16752

16752

16573

15813

31

10

7

645

67

179

179

1045

277

277

277

173

68

66

2

105

105

143

34

34

109

109

30

30

10

20

108

108

108

314

235

1

234

13

13

66

66

2205

2

2

2

24

24

21

3

164

24

13

11

140

107

33

1965

1965

1305

17

2

1

2

3

170

3

18

18

43

10

2

158

13

48

129

2

19

2

50

50

50

1983

26

26

4

22

75

74

6

42

26

1

1

121

83

15

10

25

33

38

38

1761

1761

1761

2491

379

2

2

377

166

7

153

51

2112

262

186

76

5

5

73

30

43

40

3

1

36

2

2

352

3

81

1

263

4

70

5

65

22

22

1267

1267

19

15

4

395

247

247

1

46

54

145

1

148

148

148

16

16

10

10

6

6

3019

3019

3019

3015

346

2669

4

4

9

9

9

9

9

6

6

6

6

6

6

1

1

1

1

1

1

54

54

54

54

37

32

4

1

3

3

14

14

365

365

365

360

21

21

274

78

73

23

4

3

61

32

65

65

5

1

1

4

4

35

35

35

35

35

35

127

127

127

127

1

1

1

1

1

1

124

124

411

36

28

28

28

28

8

8

8

8

68

68

68

68

68

307

307

307

173

173

134

67

67

5665

3664

288

135

135

135

80

6

6

20

1

1

18

2

2

35

35

13

13

4

4

73

1

1

72

49

19

4

19

19

9

7

2

10

10

13

13

13

13

3344

231

71

71

44

43

1

36

36

1

1

79

9

13

10

4

1

42

5

5

5

4

4

3

1

42

1

1

5

5

3

2

1

19

7

9

2

1

2

2

8

8

4

4

23

13

5

8

10

10

3

3

3

45

45

45

354

10

10

321

5

147

30

39

17

3

13

1

23

1

8

34

23

23

2328

2

2

48

48

1

1

2113

4

119

90

21

18

2

15

45

46

15

98

26

177

35

3

263

30

23

5

1

141

46

10

14

3

8

11

41

44

1

12

5

4

2

1

16

1

2

70

26

24

11

7

53

19

2

2

65

79

4

16

39

26

250

22

164

108

56

95

61

7

7

47

14

14

20

20

113

11

11

28

28

5

5

3

3

17

17

48

18

30

1

1

88

1

1

24

24

60

4

11

14

4

1

26

3

3

13

13

13

1948

186

20

18

1

1

8

1

1

6

2

2

24

1

1

8

1

7

15

13

2

28

1

1

6

6

21

3

18

34

34

5

29

64

64

3

5

7

1

10

1

2

6

1

12

8

1

1

1

5

16

16

14

2

1762

25

25

3

1

9

4

8

18

1

1

13

13

4

1

3

3

3

1

2

4

2

2

1

1

1

1

7

7

7

57

12

1

2

9

45

45

1566

16

2

1

2

11

7

7

3

3

17

17

171

3

5

6

4

1

152

35

20

15

681

44

109

16

7

4

4

1

1

1

25

3

7

7

1

1

2

9

24

11

8

32

230

7

1

2

1

2

20

12

4

5

44

1

3

2

1

22

7

629

43

192

361

7

7

9

8

2

6

6

1

1

42

19

2

2

1

14

11

11

12

1

11

1

1

1

39

34

1

33

5

5

32

32

13

13

8

5

19

1

1

7

4

3

4

4

1

1

6

6

7

7

7

2

2

2

2

2

2

1

1

14

14

14

14

14

14

14

14

14

2

2

12

12

106

106

12

12

4

4

8

8

94

94

94

82

2

3

2

1

4

1141

202

198

198

198

198

4

4

4

4

2

2

2

2

2

301

14

14

14

14

287

287

287

287

636

636

85

85

85

493

81

1

80

154

154

16

14

2

19

19

223

82

141

58

58

58

269

269

269

269

99

4

95

41

41

129

8

121
