# Supplementary material for: Ultradeep 16S rRNA Sequencing Analysis of Geographically Similar but Diverse Unexplored Marine Samples Reveal Varied Bacterial Community Composition
Source: PLoS One. 2013 Oct 22;8(10):e76724. doi: 10.1371/journal.pone.0076724 (PMC3805540; doi:10.1371/journal.pone.0076724)
Supplement: Figure S3 — Graphical representation of the relative abundance of bacterial diversity from phylum to species level of SR can be visualized in this file using Krona visualization tool. (HTML) [file pone.0076724.s003.html]

Javascript must be enabled to view this page.

members
magnitude

SR\_krona

137050

0

0

0

0

0

0

0

137050

2

2

2

2

2

2

5564

803

803

771

224

5

10

209

14

14

533

291

5

1

236

16

13

13

3

1

2

14

2

2

12

12

2

2

2

504

504

471

51

51

420

276

144

25

25

25

1

1

1

4

4

4

3

3

1

2

1225

1225

700

1

1

45

2

43

2

2

471

2

7

190

29

3

2

5

1

15

10

147

2

56

1

1

181

181

294

294

294

231

230

230

1

1

299

299

299

4

4

111

25

7

79

37

37

144

144

3

3

2733

107

107

107

6

1

7

93

2626

2625

5

5

130

130

127

10

117

3

3

7

7

15

1

14

355

24

14

317

361

361

633

365

11

57

69

18

3

60

14

36

21

21

158

158

12

6

1

5

228

20

40

124

44

2

2

26

25

1

3

3

72

72

33

33

54

54

3

3

321

321

56

56

1

1

1

148

148

148

3

3

3

111

5

5

29

29

77

71

6

25

25

25

9

1

1

8

8

8587

8397

39

39

7

5

2

32

32

1667

1667

28

1

27

4

4

1213

4

1192

17

35

35

142

70

2

70

50

8

18

6

18

38

26

12

39

1

13

25

27

27

5

4

1

44

1

43

42

42

510

11

11

11

499

499

499

3

3

2

2

1

1

33

33

8

8

7

7

6

6

12

12

260

211

8

8

15

15

12

12

19

18

1

101

101

23

11

1

11

33

5

27

1

14

14

5

9

35

2

2

33

25

4

4

5885

5885

32

32

60

60

2

1

1

855

385

16

41

413

128

3

4

11

2

108

2

2

56

2

54

3

3

4648

5

1

1115

1238

3

24

226

38

14

3

1

1

2

6

14

1474

25

59

15

32

22

25

208

20

1

76

97

97

2

2

190

190

190

190

190

10

10

2

2

2

1

1

8

8

6

1

2

3

2

2

62

62

62

62

4

4

7

7

30

15

15

21

12

9

244

210

210

210

210

210

8

8

8

8

8

26

26

26

26

26

701

701

701

701

19

19

449

449

171

1

138

27

5

62

11

1

8

21

2

19

45

45

45

45

45

45

508

508

508

508

69

12

57

273

273

119

119

47

4

28

15

6

6

6

6

6

6

7599

7599

73

73

73

73

6317

26

1

1

25

25

68

68

27

41

17

2

2

15

3

5

7

3

3

3

286

286

286

86

86

86

2

1

1

1

1

333

5

4

1

3

3

14

14

311

311

4

4

4

166

166

1

1

1

1

6

3

54

4

2

1

2

9

60

16

1

4

203

103

75

6

2

8

1

2

9

100

99

1

409

62

62

5

5

110

91

19

1

1

17

17

46

8

38

6

6

72

5

5

37

3

1

11

7

3

90

90

1517

236

182

11

1

6

3

3

29

1

3

3

1278

202

138

266

672

106

50

50

56

53

3

123

5

5

117

12

102

1

2

1

1

7

7

3

2

2

1

1

1

5

5

5

42

2

1

1

1

1

39

30

6

3

275

275

4

1

243

4

2

3

6

1

6

1

1

3

1280

1280

1280

296

296

296

190

4

2

2

186

71

1

66

8

40

21

4

1

3

17

5

12

42

37

29

8

5

5

4

4

4

21

21

2

11

8

1

1

1

30

14

14

16

16

609

31

31

186

186

392

93

4

5

274

16

10

2

1

1

8

3

5

134

134

134

9

9

9

9

664

664

652

4

121

32

272

36

54

1

110

1

1

3

17

12

12

437

437

9

3

6

97

95

2

178

92

86

93

93

60

60

99

99

38

38

61

61

4

4

4

3

3

3

1

1

1

52649

52649

52649

52649

52649

156

51287

1

16

21

50

62

53

55

4

38

1

1

6

2

31

10

855

53168

1355

2

2

1

1

1

1

308

4

4

2

1

1

299

29

3

26

2

2

268

124

114

2

19

1

8

5

5

5

1045

1045

6

6

1039

1039

7247

2080

181

181

91

90

40

40

40

315

315

291

24

244

13

6

7

177

166

11

54

52

2

1

1

1

88

16

16

71

17

11

5

1

37

1

1

28

23

23

5

5

68

3

3

52

52

9

1

8

4

4

10

10

10

823

6

4

2

4

2

2

3

3

590

590

220

220

58

58

50

8

202

6

6

192

192

4

1

1

2

22

22

22

88

43

43

43

16

16

16

5

3

3

2

2

24

13

3

10

5

5

6

3

3

1015

921

652

652

4

4

123

123

8

8

10

3

7

124

1

123

94

2

2

20

20

1

1

3

3

2

2

65

2

5

2

56

1

1

28

28

15

15

13

8

5

2089

2089

2088

59

2029

1

1

369

369

75

75

22

12

9

1

46

46

226

222

4

1578

51

23

23

18

18

10

10

1

1

1

1526

210

210

1

1

2

1

1

1

1

48

48

126

52

33

3

38

10

10

8

8

10

10

4

4

12

12

456

322

31

1

102

88

85

2

1

108

3

10

1

86

8

121

114

7

49

49

5

5

215

215

1

1

50

22

7

11

1

9

1

1

17665

12

12

2

2

10

5

4

1

38

38

16

4

1

5

6

22

22

9

9

8

8

1

1

1855

1855

5

3

2

3

3

7

7

6

6

1834

1834

199

9

9

6

3

44

44

18

14

12

44

44

44

70

70

1

53

1

15

32

5

2

3

6

6

3

1

2

7

7

11

9

2

767

486

486

452

26

4

2

2

281

246

244

2

35

35

267

267

138

133

4

1

2

2

1

1

4

4

106

1

2

1

2

7

1

2

19

1

1

1

4

3

2

2

3

1

1

41

11

16

16

7321

7321

30

28

2

3

3

1

1

7285

2

47

3

11

10

3

17

77

70

54

1

37

2

81

174

6678

1

1

16

1

1

1

1

1567

1567

150

1

148

1

18

18

1

1

1398

1398

165

3

3

1

1

1

30

30

29

1

132

4

4

2

2

126

125

1

3234

2003

1947

1947

36

36

12

1

11

8

4

4

500

117

4

113

2

2

114

1

1

112

14

14

138

50

88

17

17

14

3

3

8

40

1

37

1

1

18

18

1

1

24

24

1

1

731

731

479

252

1409

1409

28

28

8

8

9

6

3

5

5

6

6

5

5

15

5

9

1

1

1

153

152

1

486

458

27

1

5

1

4

516

195

6

1

312

1

1

15

15

155

2

153

2

2

150

76

76

76

17

17

12

5

24

24

3

9

12

10

10

10

23

23

23

672

252

7

7

245

9

117

41

50

3

17

1

2

1

1

3

420

5

4

1

415

20

1

62

270

33

8

21

713

556

85

40

37

3

43

41

1

1

2

2

71

61

22

22

4

13

7

6

1

3

1

2

12

6

6

3

3

1

1

1

1

1

1

358

5

5

156

156

2

2

195

195

30

2

2

1

1

1

1

5

5

7

7

9

7

1

1

2

2

3

2

1

89

89

1

1

21

21

67

25

42

37

37

15

15

1

1

3

3

3

2

1

1

1

14

1

1

7

5

22

22

22

1

21

7

7

7

7

2

2

2

1

1

23565

16435

16435

16381

15920

83

2

31

311

34

54

54

347

20

20

20

69

45

45

24

24

8

8

8

12

12

3

9

86

86

86

152

53

53

99

99

1668

25

25

25

1

1

1

45

15

12

3

30

24

6

1497

5

5

1492

496

36

1

18

2

2

104

1

16

17

17

27

16

3

493

21

33

29

15

145

100

100

100

709

166

1

1

165

84

81

104

104

41

63

169

164

9

1

4

2

1

58

89

5

5

270

270

270

4243

148

3

3

136

86

33

17

9

3

6

4095

365

274

91

9

9

125

30

95

167

4

1

162

3

3

499

2

199

1

297

59

10

49

163

163

38

30

3

1

4

4

4

2601

2601

62

59

3

156

99

99

1

1

42

37

18

57

57

57

7

1

1

1

6

6

6

2617

2617

2617

2611

629

1982

6

6

6

6

6

6

6

1

1

1

1

1

1

1

1

1

1

1

1

102

102

102

102

78

53

25

3

3

21

21

597

597

597

593

2

2

477

147

3

36

3

15

16

59

5

193

114

5

107

2

4

4

4

6

6

6

6

6

6

14

14

14

14

1

1

1

1

6

6

6

6

736

27

20

20

20

20

7

7

7

7

3

3

3

3

3

8

2

2

2

2

6

6

6

6

698

698

698

674

674

24

12

12

4856

3485

355

95

95

95

153

4

4

25

2

1

20

2

4

4

46

46

50

50

20

20

4

4

107

107

87

6

14

9

9

9

9

62

62

3

3

11

11

48

48

7

7

7

7

3052

152

43

43

20

20

11

11

2

2

76

8

6

17

1

44

4

4

4

63

4

4

59

58

1

68

2

2

50

32

11

4

3

12

12

3

3

1

1

60

48

13

35

12

12

22

22

22

159

31

31

117

19

32

36

3

3

4

6

3

2

1

8

11

11

1876

3

3

71

71

3

3

1757

1

19

2

10

6

2

18

24

21

163

133

111

6

1

3

3

117

4

19

29

4

168

6

55

17

25

3

8

7

63

15

9

1

4

18

1

51

9

3

12

13

73

18

50

3

94

19

85

10

4

21

33

21

19

123

42

6

36

39

20

10

8

2

18

18

1

1

111

2

1

1

5

5

1

1

19

4

15

35

35

26

22

4

1

1

22

22

1

1

1

497

22

22

18

1

17

457

6

9

231

4

4

196

1

6

1300

124

7

6

5

1

1

1

8

8

6

2

22

3

3

10

10

9

9

21

21

1

5

15

49

49

5

6

1

5

1

1

13

1

4

12

17

1

1

16

10

6

1176

61

61

2

6

1

5

2

9

3

31

2

8

4

4

4

2

2

8

8

8

2

1

1

1

1

5

5

5

55

29

1

1

21

2

4

26

26

825

40

2

38

1

1

9

9

135

3

2

2

2

126

6

3

3

501

1

2

2

38

11

6

3

5

13

1

57

5

1

9

15

1

3

1

16

4

3

36

7

11

33

2

11

40

3

2

29

22

8

14

44

1

26

1

9

1

1

3

133

1

13

95

4

1

10

9

105

80

5

3

1

7

4

60

8

8

17

2

7

6

2

2

2

2

105

9

9

15

15

81

80

1

45

45

4

4

4

41

3

3

4

4

2

2

3

3

21

11

10

4

4

3

3

1

1

26

26

26

21

5

16

3

3

2

2

69

69

69

69

69

69

49

49

23

23

2

2

21

21

26

26

26

14

10

2

412

71

68

68

68

68

3

3

3

3

123

18

18

18

18

105

105

105

105

218

218

5

5

5

198

44

1

43

20

20

20

18

2

3

3

111

13

98

15

15

15

910

910

910

910

590

2

588

16

5

4

1

1

5

304

138

166
