# Supplementary material for: Ultradeep 16S rRNA Sequencing Analysis of Geographically Similar but Diverse Unexplored Marine Samples Reveal Varied Bacterial Community Composition
Source: PLoS One. 2013 Oct 22;8(10):e76724. doi: 10.1371/journal.pone.0076724 (PMC3805540; doi:10.1371/journal.pone.0076724)
Supplement: Figure S4 — Graphical representation of the relative abundance of bacterial diversity from phylum to species level of SW can be visualized in this file using Krona visualization tool. (HTML) [file pone.0076724.s004.html]

Javascript must be enabled to view this page.

members
magnitude

SW\_krona

173150

0

0

0

0

0

0

0

173150

1

1

1

1

1

1

14831

454

454

398

195

8

11

176

34

34

169

148

17

1

3

49

43

42

1

6

6

5

3

3

2

2

2

2

2

358

358

308

35

35

273

218

55

15

15

15

9

6

6

3

3

26

26

1

1

7

1

1

1

3

1

3

1

3

3

2063

2063

1888

3

3

12

2

8

2

1

1

12

12

4

1

3

1780

34

370

168

24

159

464

1

284

36

23

52

1

159

5

76

76

92

92

92

83

81

81

2

2

163

163

163

86

16

70

22

6

9

7

47

47

8

8

11793

919

919

919

884

9

26

10874

10870

52

52

186

186

300

2

12

286

199

199

14

13

1

41

29

12

3

3

967

766

143

58

183

183

1697

709

3

1

30

8

198

10

316

369

25

28

282

282

3045

3045

616

3

19

422

1

161

10

1632

53

1485

59

11

24

27

27

2

2

132

132

6

3

3

13

1

12

1239

1239

9

9

15

15

1

1

84

84

68

68

57

57

4

4

4

164

164

164

78

2

2

13

13

63

47

16

10

10

2

2

2

2

2

13

13

13

63

1

1

24

24

38

38

542

541

17

17

4

4

13

13

129

129

27

1

26

38

38

22

1

1

13

7

1

1

3

1

1

1

5

5

4

1

3

1

1

27

25

2

1

1

19

19

19

19

74

12

8

8

4

4

62

62

3

45

14

302

302

3

3

281

1

10

4

5

89

98

7

1

1

21

4

7

1

4

16

7

5

4

4

6

3

3

8

8

1

1

1

1

1

137

137

3

3

3

2

1

134

134

134

2

1

22

98

1

10

7

7

7

7

1

1

2

2

4

4

7

3

3

3

3

3

4

4

4

4

4

16

16

16

16

6

1

5

10

5

1

4

49

49

49

49

11

11

13

13

25

25

13786

13786

1

1

1

1

13423

1

1

1

64

64

46

18

15

15

1

14

3

3

3

43

43

43

11

11

11

5

4

4

1

1

14

1

1

1

1

12

12

6

6

6

407

407

1

3

3

2

2

7

1

4

1

1

36

14

1

2

1

4

5

62

1

5

98

1

3

3

3

4

2

39

12

3

5

12

5

27

32

2

51

51

1

1

3

2

3

17

5

1

3

1

14

5924

2269

2269

1

1

38

38

114

113

1

8

8

1

1

763

763

58

47

8

3

2031

6

8

701

52

4

2

310

1

8

1

4

42

225

6

35

625

1

620

620

14

14

7

7

7

7

7

231

9

4

4

1

2

1

1

5

5

215

64

50

1

86

14

51

32

32

13

13

6

3

3

413

379

4

358

17

32

2

30

2

2

115

115

16

3

10

47

34

5

1

1

1

243

7

2

5

4

4

177

43

134

55

35

3

2

4

3

7

1

576

576

1

10

1

484

1

5

1

4

8

43

3

2

2

1

3

1

5

1

32

32

32

2006

2006

2006

8

2

1

1

6

2

1

3

1926

502

4

498

1424

198

1226

19

12

1

11

7

7

36

36

12

6

1

14

3

4

4

3

1

30

24

24

6

6

1153

380

380

58

58

715

441

8

138

128

17

6

6

11

2

1

3

4

1

2

2

2

9

9

9

1

1

1

1

46

46

44

3

7

7

11

8

8

2

2

313

313

1

1

20

12

5

3

8

8

284

284

2

2

2

2

58516

58516

58516

58516

58516

2

55498

24

189

12

160

1

6

261

1

4

40

17

12

4

2

14

17

10

29

2213

69698

49

30

1

1

1

29

11

1

10

18

10

1

1

6

19

19

19

19

37509

929

645

580

1

579

12

12

9

7

2

44

44

284

6

3

3

50

50

1

1

1

1

25

25

177

4

46

33

94

24

24

18953

18953

18

18

18935

22

1

18912

875

860

13

13

136

136

51

14

31

6

269

269

391

1

7

2

379

2

15

6

6

9

5

4

9242

1726

1726

321

2

18

1385

222

222

222

232

232

183

49

31

13

11

2

17

15

2

1

1

77

36

36

36

9

14

3

10

2

2

3

3

141

141

141

58

5

5

3

3

50

3

47

63

1

1

62

62

5415

95

23

6

4

62

30

30

65

65

4746

4746

479

479

147

147

88

57

1

1

1014

14

14

948

948

52

2

1

49

116

116

116

17

17

5

5

1

1

11

11

335

6

6

6

122

122

122

68

1

1

2

1

1

49

49

16

16

139

84

29

55

9

9

10

9

1

29

29

7

7

8

8

8

8

7150

653

328

328

25

25

244

233

11

56

56

48

48

12

1

35

6449

1216

1216

26

3

3

20

16

16

22

22

440

440

865

1

4

660

85

1

7

107

1

1

23

21

2

178

148

30

28

28

1079

353

167

22

17

15

505

25

25

464

1

7

6

5

7

1

9

426

2

13

13

1066

46

44

14

929

12

19

2

336

1

314

21

36

36

2

2

4

4

34

34

80

80

155

155

14

14

10

10

1

1

259

2

25

91

5

17

1

118

31

31

6

6

19

19

26370

185

185

4

3

1

1

1

180

1

175

2

2

380

380

27

5

2

1

1

17

1

353

352

1

30

30

30

30

152

152

7

7

1

1

12

12

132

132

2779

857

857

1

10

350

8

10

6

3

3

461

5

59

59

59

424

424

3

2

329

16

2

72

66

66

39

27

1373

3

3

602

40

1

27

534

446

87

1

20

338

278

278

4

4

34

34

6

6

1597

1254

1254

1234

4

3

4

9

343

133

132

1

210

210

5322

5322

3074

1

3014

55

2

2

10

9

1

2

2

2221

1

77

31

39

40

6

105

1

4

101

1

3

5

7

1

1

10

36

29

14

448

30

1

3

8

1

2

9

32

1

14

3

2

1

57

3

2

4

12

1

2

23

11

19

31

1

608

6

6

1

1

359

7

15

15

2

2

2

2

7867

7867

1

1

8

8

7858

1

12

3

5

1

253

70

18

8

9

209

1

7

1

7236

4

11

9

732

732

609

1

2

1

173

421

6

5

45

37

8

1

1

77

77

123

80

80

43

1

1

9

26

6

6

4

2

37

13

13

1

1

23

23

420

208

206

206

2

2

96

13

13

83

83

116

1

1

115

82

33

1881

1881

2

2

16

16

6

6

29

22

2

4

1

9

9

1038

7

1030

1

13

5

8

13

13

32

22

6

4

28

2

9

14

2

1

6

6

9

9

448

430

17

1

212

8

8

3

1

146

46

9

9

2

2

9

6

2

1

2165

206

206

206

600

13

13

6

3

3

581

2

3

50

464

21

1

1

32

7

1112

14

14

1

1

1097

122

354

621

201

201

2

1

34

2

25

2

135

46

46

46

2735

1488

206

206

1282

35

791

13

90

121

4

20

142

25

2

10

7

1

1

19

1

1247

9

2

6

1

5

5

1233

13

3

1

32

2

87

1

1

74

94

8

15

294

304

1

41

2

23

237

2213

999

74

15

5

2

8

50

32

18

9

6

3

235

7

7

182

39

16

1

8

11

86

5

5

2

8

1

1

1

45

19

2

22

1

1

56

48

48

4

4

2

2

2

2

151

30

30

3

3

78

78

2

2

38

38

483

46

46

1

1

11

11

2

2

22

17

1

4

357

172

163

22

22

22

20

4

16

2

2

401

401

110

110

291

141

150

56

56

16

16

1

1

22

19

3

2

1

1

15

5

1

2

1

1

5

520

520

520

416

104

234

207

207

207

27

1

1

26

2

24

3

3

3

3

1286

825

825

811

802

1

8

14

14

128

5

5

5

23

5

3

2

18

18

87

31

31

56

56

8

8

8

2

2

2

3

3

3

165

5

5

5

29

9

8

1

20

20

121

2

2

119

64

17

3

6

3

2

1

13

3

5

2

10

10

10

37

8

2

2

6

6

2

1

1

1

1

27

27

27

67

8

8

4

4

59

1

1

1

1

2

2

13

10

3

8

5

3

2

2

1

1

13

13

10

10

8

8

62

40

40

1

8

31

22

22

22

2

2

2

2

2268

2268

2268

2268

1457

811

3

3

3

3

3

15

15

15

15

1

1

10

10

4

1

3

7

7

7

7

7

7

7

7

7

7

1

1

6

6

13

6

1

1

1

1

5

4

4

1

3

1

1

1

7

7

7

7

7

5957

510

55

36

4

4

3

3

26

26

2

2

1

1

19

15

15

4

3

1

5

5

1

1

4

4

3

3

3

3

447

29

8

8

5

5

12

12

4

2

2

13

13

12

1

20

1

1

13

3

4

3

3

5

5

1

1

4

2

2

2

2

1

1

1

14

14

14

79

67

11

1

15

21

2

5

2

2

4

4

12

12

231

2

2

225

11

26

3

1

8

2

1

10

2

2

7

21

3

1

4

17

6

1

2

1

1

8

12

2

5

2

2

2

12

3

4

8

6

29

4

3

1

42

20

20

1

1

7

2

5

2

2

2

1

1

4

4

4

3

1

2

2

14

11

3

2

2

1

1

1

1

3

3

5374

231

15

13

3

1

7

1

1

2

1

1

12

4

4

8

1

6

1

40

21

2

19

18

13

5

1

1

24

8

8

16

1

3

1

9

2

117

115

1

3

1

2

21

3

3

3

1

2

2

5

3

2

1

14

1

4

1

42

2

2

23

8

8

15

11

4

5143

173

2

2

171

17

4

8

4

2

1

1

1

3

21

1

34

57

3

14

164

15

14

1

149

2

93

4

50

12

7

7

4

4

1

1

515

87

1

35

1

2

29

1

1

6

3

3

5

428

70

5

13

8

36

199

97

4213

52

2

5

45

3

3

72

6

65

1

673

562

17

6

88

376

117

1

2

215

8

3

1

11

9

1

8

20

19

1

2922

2

7

21

5

2

497

10

53

17

6

1

9

13

5

1

2

91

3

3

9

1

1659

11

9

7

11

2

45

3

7

23

91

3

188

7

1

5

63

29

86

29

52

5

1

1

8

8

35

24

23

1

11

11

2

2

2

22

7

3

4

15

2

13

7

1

1

6

6

62

62

25

25

25

37

1

1

1

1

19

17

1

1

1

1

9

1

8

4

3

1

2

2

11

11

11

2

1

1

4

4

3

3

2

2

604

604

551

551

551

545

6

53

53

53

20

2

4

2

12

1

1

10

1

8782

8234

8234

8234

8234

8234

47

37

37

37

37

10

10

10

6

4

501

501

22

22

22

479

18

18

343

343

38

38

3

3

77

26

51

11

11

11

11

6

6

5

2

3
