# Supplementary material for: Ultradeep 16S rRNA Sequencing Analysis of Geographically Similar but Diverse Unexplored Marine Samples Reveal Varied Bacterial Community Composition
Source: PLoS One. 2013 Oct 22;8(10):e76724. doi: 10.1371/journal.pone.0076724 (PMC3805540; doi:10.1371/journal.pone.0076724)
Supplement: Figure S5 — Graphical representation of the relative abundance of bacterial diversity from phylum to species level of SG can be visualized in this file using Krona visualization tool. (HTML) [file pone.0076724.s005.html]

Javascript must be enabled to view this page.

members
magnitude

Repl\_SG\_krona

223953

0

0

0

0

0

0

0

223953

4

4

4

4

4

4

16670

1748

1748

1652

304

8

16

2

278

163

163

1185

740

12

1

432

56

34

34

22

16

6

38

1

1

37

37

2

2

2

2779

2779

2566

2155

2155

411

342

69

24

24

24

6

2

2

4

2

2

175

175

4

91

9

2

15

9

28

14

2

1

8

8

1

1

3

3

3081

3081

1721

3

3

5

5

84

3

1

1

78

1

2

2

21

21

35

2

33

1265

7

6

43

359

52

2

29

1

66

300

44

165

29

124

27

11

306

306

403

403

403

957

953

953

4

4

1006

1006

1006

9

9

670

437

231

2

37

37

231

231

59

59

8056

157

157

157

6

13

138

7899

7899

84

84

771

771

1

1

225

8

217

2

2

62

43

19

53

18

35

953

72

68

813

1430

1430

1081

239

88

53

118

4

5

212

344

18

1

1

474

474

175

8

103

59

5

1547

552

26

890

19

60

7

7

1

1

56

56

24

12

12

2

2

90

90

35

35

102

102

4

1

3

13

13

669

669

37

37

137

137

137

100

3

3

13

13

84

54

30

10

10

2

2

2

2

2

7

7

7

20

4

4

13

13

3

3

7587

7462

112

112

21

9

12

91

91

1364

1364

40

16

24

39

33

1

5

708

6

3

536

163

6

6

100

50

50

100

39

13

39

6

3

5

4

1

37

1

14

22

36

36

53

30

3

10

3

7

205

1

51

153

35

35

591

19

19

19

572

572

572

1

1

1

1

70

70

1

1

13

13

41

41

15

3

12

521

308

11

11

76

76

77

77

14

2

5

5

2

77

77

4

4

38

23

2

2

11

11

1

8

2

4

4

4

209

3

3

206

156

46

4

4803

4803

14

14

2

2

2

2

623

287

6

26

304

102

1

2

10

4

85

60

2

38

20

13

13

3905

12

2

735

820

50

42

276

27

97

1

2

30

1311

2

35

1

115

29

3

1

35

147

39

93

69

4

52

13

13

13

125

125

125

125

125

31

31

20

20

20

1

12

3

4

4

4

4

1

2

1

7

7

6

1

5

1

1

129

129

129

129

5

5

117

107

2

8

7

7

72

72

72

72

72

72

62

62

62

62

5

5

1

1

40

40

8

8

8

1

3

1

3

1

1

1

1

1

1

318

318

318

318

4

1

3

101

101

90

90

123

120

3

9

9

9

9

8

8

1

1

20097

20097

28

28

28

28

15985

185

3

3

2

2

81

2

65

14

99

75

22

2

24

24

24

184

184

41

143

197

197

2

4

148

4

7

2

17

7

1

2

2

1

1

1

1

5

5

5

84

84

84

289

287

287

2

2

2339

2339

2339

71

40

40

31

31

235

3

3

232

40

2

181

4

5

60

9

9

51

8

43

132

80

53

27

52

52

1

1

1

701

3

1

1

1

23

7

16

6

6

1

1

4

4

664

664

8

8

8

698

698

1

8

1

1

1

28

5

4

38

3

1

7

1

56

423

120

188

101

43

2

1

23

1

16

15

87

79

5

3

52

52

6

1

2

1

4

38

389

61

61

5

5

15

15

1

1

78

78

57

57

14

14

99

1

12

2

30

10

17

1

22

4

59

59

5153

98

82

1

1

2

12

5055

2299

723

1781

3

249

11

11

11

306

13

13

293

293

646

440

440

203

191

12

3

3

39

39

2

6

1

1

5

3

21

2528

178

178

37

37

2313

215

6

2062

30

1243

1

1

1242

2

6

1232

1

1

13

13

4

6

3

200

200

200

3

3

3

11

11

11

11

3218

3218

3195

585

1

417

1615

22

38

221

19

76

4

41

21

1

2

132

23

23

803

803

4

3

1

5

2

3

682

167

514

1

43

43

69

69

52

52

52

52

72910

72910

72910

72910

72910

65

70116

3

155

494

234

1

3

94

6

125

1

22

6

175

6

184

1220

91242

1471

2

2

1

1

1

1

314

1

1

1

312

68

68

11

11

1

1

232

124

36

38

3

31

1

1

1

1155

1155

2

2

1153

1153

41905

1102

701

350

350

21

20

1

155

155

40

40

7

6

1

128

16

112

401

12

3

3

3

3

119

119

220

10

101

109

17

17

5

2

1

2

5

5

19

19

4

4

14946

14946

15

15

14925

5

47

14870

3

6

6

1319

1291

1

1

451

451

74

15

37

21

1

381

1

378

2

384

13

2

369

28

7

7

21

1

11

9

10561

1476

1476

390

2

25

1059

340

340

339

1

863

862

4

780

9

69

1

1

143

1

1

69

53

1

13

2

73

66

7

3

3

3

551

71

71

459

1

36

1

3

226

2

2

5

10

127

40

4

2

21

21

1130

17

17

2

2

1106

3

1

1102

1

1

4

4

151

2

2

69

69

80

80

106

106

105

1

2820

119

9

6

8

96

36

36

29

29

1030

1030

1606

1606

785

785

221

564

1780

1713

1712

1

67

51

16

413

413

413

43

43

1

1

4

1

3

38

8

27

2

1

230

40

3

2

1

37

37

13

1

1

12

12

73

13

13

1

1

59

59

104

8

5

3

8

8

13

4

7

2

71

68

2

1

4

1

3

4

4

4

4

13700

747

66

66

1

1

535

526

9

145

145

17

17

2

4

1

10

12936

1951

1951

51

3

2

38

6

2

1

1

37

37

318

318

721

1

1

19

370

65

4

20

241

244

166

78

36

36

12

12

7

6

1

3160

1677

204

10

2

16

1251

1481

1

20

4

1

1411

42

2

87

87

664

5

16

56

2

4

70

498

13

1

1

4

4

268

268

10

10

1194

2

404

318

341

129

22

22

6

1

5

2

2

25

25

12

10

2

45

45

1411

1403

8

12

1

11

99

99

985

985

3

3

1

1

66

66

22453

65

65

4

4

3

1

2

13

13

45

41

3

1

327

326

265

42

12

1

32

32

146

61

61

1

1

1

9

9

9

9

1993

7

7

7

1986

1

1

2

1

1

2

2

3

1

2

8

8

1970

1970

344

1

1

1

1

1

1

108

108

1

71

9

27

15

15

15

184

184

176

5

3

35

2

2

4

1

2

1

4

2

2

17

17

4

4

4

4

1902

1440

1440

1317

2

3

11

1

1

2

3

49

1

36

1

2

11

462

413

413

49

49

872

872

632

1

476

140

15

3

1

2

10

10

203

1

1

12

1

2

1

2

12

5

4

4

1

2

1

6

6

1

3

4

2

8

2

1

9

1

9

3

59

1

1

38

3

3

21

21

7423

5

5

5

7418

3

3

44

42

2

4

4

4

4

7363

2

1

19

5

63

1

2

59

4

169

19

11

26

202

89

6657

7

26

1

2638

2638

257

250

2

5

21

21

2354

2354

6

6

345

58

58

2

3

39

10

2

2

16

16

11

4

1

271

2

2

6

6

263

263

3238

1316

2

2

1282

1282

1

1

18

18

11

4

2

5

2

1

1

593

58

8

50

3

3

337

2

335

9

9

47

35

12

9

9

2

2

33

32

1

87

87

1

1

5

5

2

2

1329

1329

958

370

1

1933

1933

1

1

1

1

6

6

293

21

169

102

1

485

262

1

147

2

4

10

53

6

8

7

1

499

65

1

429

2

2

1

1

17

17

12

12

23

23

3

1

2

32

5

3

24

5

3

2

21

1

18

2

1

1

474

3

442

29

29

9

9

11

3

2

1

9

9

10

10

516

183

183

183

12

2

2

10

1

3

6

88

70

54

8

8

11

11

7

7

97

97

97

136

136

136

848

348

4

4

344

6

1

255

9

43

6

10

9

3

2

500

3

3

6

6

491

9

17

9

125

145

13

6

105

2

13

15

32

4370

1321

5

1

1

2

2

2

2

342

309

169

4

42

74

2

1

12

5

2

2

1

1

30

9

21

103

55

3

52

6

6

12

12

1

1

29

29

604

46

46

271

271

1

1

7

7

279

279

267

1

1

37

37

5

3

2

2

2

32

32

55

4

51

65

4

43

14

4

39

10

18

2

9

9

9

10

1

9

10

10

2

2

504

504

20

20

4

4

108

108

328

125

203

44

44

1155

1155

117

117

12

12

39

39

45

22

19

4

52

39

13

18

18

872

37

20

128

9

2

25

6

100

545

1347

1347

1319

2

1317

28

28

18

15

15

15

3

3

3

25

25

25

25

19681

15476

15476

15248

15204

8

1

18

2

15

228

228

306

21

21

21

48

22

12

10

26

26

135

91

91

44

44

12

12

12

4

4

4

86

43

43

43

42

1

1190

1

1

1

25

25

4

21

297

211

207

4

86

82

4

745

2

2

743

192

75

1

2

6

8

58

2

31

116

44

45

5

55

6

2

13

8

35

6

2

22

9

122

122

122

275

4

2

2

2

2

111

109

19

39

51

2

2

49

46

4

42

3

3

111

111

111

2028

449

38

38

29

29

1

1

256

168

56

32

125

2

123

1579

221

206

15

10

10

35

6

29

9

1

8

6

6

389

102

286

1

40

1

39

8

8

6

4

2

1

1

763

763

91

88

3

400

113

113

7

32

23

47

3

1

287

287

286

1

6

6

6

6

1315

1315

1315

1309

168

1141

6

6

47

47

47

47

47

3

3

3

3

3

3

16

16

16

16

9

6

2

1

3

3

4

4

2035

2035

2035

2034

10

6

4

1097

6

5

3

5

25

10

2

79

908

54

927

917

10

1

1

1

2

2

2

2

2

2

10

10

10

10

4

4

2

2

4

4

310

9

4

4

4

4

5

5

5

5

279

279

279

279

4

274

1

22

22

22

4

4

18

9

9

7790

5876

748

1

1

1

195

12

12

35

1

34

127

127

15

15

4

4

2

2

552

1

1

551

491

12

47

1

15

15

15

15

51

51

29

29

6

5

1

16

16

5

5

5

5

5057

1086

584

584

185

185

29

29

7

7

3

3

278

86

10

17

9

2

2

152

2

1

1

1

1

56

1

1

5

5

29

2

27

19

19

2

2

175

77

8

69

48

3

2

43

50

3

21

20

6

6

6

6

54

54

54

274

262

3

28

1

11

10

4

25

3

125

3

7

4

7

31

12

12

2041

7

7

129

129

1868

2

77

9

13

10

1

35

18

39

1

14

27

1

31

25

2

16

1

28

419

40

8

2

30

3

25

4

4

56

6

75

81

25

5

1

25

1

3

8

28

5

1

63

7

80

1

33

9

39

2

9

39

4

111

20

4

16

12

43

27

144

37

14

23

36

34

2

1

1

30

1

1

1

1

708

1

1

18

12

6

3

2

1

46

18

28

12

12

2

2

37

37

13

2

2

9

496

83

413

76

76

4

4

1

1

1

618

12

12

12

5

7

593

120

44

192

8

208

10

10

1

1

1

1292

202

17

13

4

5

4

4

4

44

25

25

19

10

9

20

7

7

11

1

6

4

2

2

13

13

4

1

1

7

99

96

1

7

1

7

1

2

1

15

21

1

3

5

6

1

5

19

3

1

1

1

9

9

7

2

1090

98

98

7

2

1

12

1

1

3

61

7

3

4

2

2

2

1

1

4

1

1

2

1

1

1

1

36

14

1

2

1

4

6

1

1

21

4

13

1

3

804

17

12

5

2

2

1

1

14

13

1

57

1

1

1

1

53

47

34

13

484

7

3

31

4

2

12

14

50

1

12

5

1

2

3

1

1

3

12

1

23

7

28

67

61

2

1

1

35

9

4

32

2

1

31

1

4

10

177

75

93

5

3

1

5

5

29

19

3

3

3

1

1

8

10

10

3

3

3

112

2

2

31

16

15

79

79

602

602

7

7

6

1

595

5

5

380

27

353

6

3

3

28

28

144

144

25

8

17

6

1

5

1

1

15

15

15

3

1

2

8

8

4

4

5

5

5

5

5

7

7

7

7

7

7

59

59

31

31

28

28

3

3

28

28

28

19

3

1

2

2

1

4332

164

154

154

154

154

10

10

10

4

6

833

801

801

801

801

32

32

32

32

3335

3335

9

9

9

3326

684

1

3

680

317

317

802

801

1

14

14

1509

720

789

120

120

120

120

74

5

69

2

2

10

10

34

1

33
