# Supplementary figures and images for: Ultradeep 16S rRNA Sequencing Analysis of Geographically Similar but Diverse Unexplored Marine Samples Reveal Varied Bacterial Community Composition
Source: PLoS One. 2013 Oct 22;8(10):e76724. doi: 10.1371/journal.pone.0076724 (PMC3805540; doi:10.1371/journal.pone.0076724)

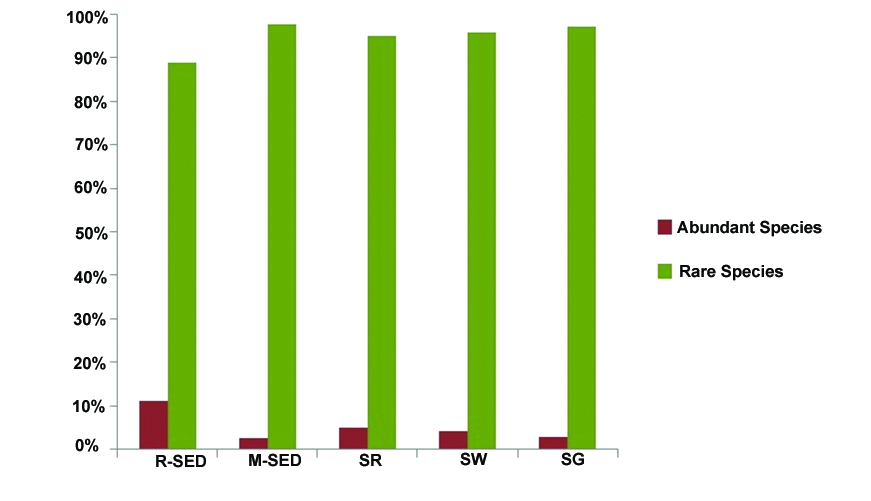

Supplement: Figure S6 — This file is a bar graph describing the percentage of abundant and rare species among the unique species of the five marine samples. (TIF) [file pone.0076724.s006.tif]
